# Hæmorrhagic Malarial Fever

**Published:** 1872-06

**Authors:** B. M. Cromwell

**Affiliations:** Albany, Georgia


### HÆMORRHAGIC MALARIAL FEVER.

BY B. M. CROMWELL, M.D., ALBANY, GEORGIA.*

A few days ago you expressed a desire to have my views on hæmaturic fever, and as I see, by a recent publication, that you are engaged in making analyses in this and kindred affections, I take the liberty of sending you this paper—written *currente calamo*, and intended to be suggestive only. Indeed, deprived as I have been of the use of a good microscope and other means of making critical examinations, I fear you will find my views but crude hypotheses; they are taken, however, from close observation at the bed-side—the place from whence, at least, all of our most valuable information comes.

I have seen a number of these cases in connection with other physicians; I have treated only three exclusive of these. The first (and, so far as I am aware, the only case up to that time that had occurred here) was a white boy aged about ten years. He had been suffering all the summer with chronic chills—was anæmic, and had been treated at home. I was sent for because he was passing blood with his urine. I entirely underestimated the gravity of the symptoms, and regarding the hemorrhage as accidental and due to debility, I put him upon muriated tincture of iron and quinine in as large doses as could be borne. The treatment, as I look back at it, was correct, but the views that prompted it were erroneous, and his recovery may be regarded as accidental.

The next case was that of a mulatto brick-layer, who, while plying his vocation in the hot sun, was suddenly seized with pain in the head, and vomiting, followed by a rigor of over an hour's duration. After the rigor passed off, he began vomiting blood, and passing it at stool in such quanties as to threaten extinction of life. What my treatment was I do not now remember; but it was eclectic, and, therefore, probably injudicious. He recovered, however.

The other and last case that came under my sole management was that of a young Englishman recently arrived in this

*Through Wm. Abram Love, M.D., Atlanta, Georgia.

country. He had been having chills all summer, and was proverbially careless of his health. At the time of his attack he was seized also with great pain in the head and vomiting (I think), followed by a rigor of more than an hour. When this passed away, he began passing blood from his bladder in large quantities. Incessant vomiting throughout the whole attack, high fever, with little or no abatement from the beginning, a parched and icteroid skin, insatiable thirst, and great prostration, were the prominent symptoms all through the attack. What and how much I did for him, I can not now tell; I did all I could think of, and that was a great deal, I assure you. It was surprising how his sturdy English constitution withstood the assaults of malaria, even assisted by medical treatment. One event in the progress of his case is worthy of mention: At the closing scene, as I thought—when I had to place my ear close to his mouth to catch his almost inarticulate entreaties for help—it occurred to me to make one more effort to check the vomiting, and procure for him time to sleep; so I gave him thirty grains of calomel as a sedative. A few minutes after taking it, he had one more tremendous vomiting spell, and then fell back on his pillow, I thought, dead; but he did not die, nor did he vomit any more. After sleeping for four or five hours, he awoke, and, though in a feeble condition, slowly convalesced. He subsequently recovered his health perfectly, but his jaw-bone, I am told, suffered.

The predisposing cause of this disease is, so far as my observation extends, a previous habit of chills, and the consequent depression of the vital energies; the immediate cause, the imbibition of an inordinate dose of malaria. The prominent symptoms are: severe rigors of prolonged duration, intense pain in the head, incessant vomiting, an icteroid and parched skin, and hæmaturia.

All the cases attacked were whites expect one—the case recorded above—and it is worthy of note, as a singular coincidence, that with the advent of this new development of malarial fever, the old-fashioned congestive chill seems to have passed away; we hear nothing of it now in this section of country.

Now as to the pathology of hæmaturic fever. I regard the disease as a blood-poison, produced by the imbibition of an inordinate dose (if I may say so) of malaria—this poison producing results the same in kind, but differing in degree and intensity, as does the same agent which, under ordinary circumstances, induces only intermittent or bilious fever. Let me illustrate: The same morbific agent which ordinarily induces an attack of scarlatina simplex will, in its concentrated form, produce scarlatina maligna; and in both pathological conditions—*i. e.*, hæmaturic and malignant scarlet fever—the *processes* are the same, though the inducing agents may essentially differ. Hæmaturic fever, then, is to intermittent fever what scarlatina maligna is to the simple variety of that disease.

From many years' observation of malarial diseases, I am convinced that the blood primarily suffers in all malarial poisoning; and it suffers by reason of the death of its disks, its oxygen-carriers. This is manifest by the chlorotic appearance of all who suffer from its influence, whether acute or chronic, and by the great prostration of the nervous system that supervenes upon an attack; for it is to be remembered, that the functional activity of the nervous and muscular tissues is dependent upon their proper oxygenation, and this condition depends, first, upon a free supply of oxygen furnished in the lungs, and, secondly, upon the prompt transmission of the oxygen to all the tissues of the body.

In the milder forms of malarial poisoning, the death of the disks takes place, *pari passu*, with the severity of the attack, and the disintegrated disks are eliminated by the liver, and perhaps the kidneys, so rapidly as not to make their presence known to the casual observe. If, therefore, I am correct in supposing that death of the blood disks is the primary and chief pathological lesion in all malarial poisoning, it will be readily understood how, when a large per cent. (probably a majority) of the disks die, the capacity of the blood to transport oxygen is so reduced that the functions of the body, nutritive and eliminative, almost cease; and as the larger portion of the blood disks are no longer of any use to the economy, they are to be eliminated as effete matter—the rapidity and facility with which this is accomplished being one of the chief points of inquiry, as on it depends, in a great measure, our prognosis of the case.

What is known as "bloody urine" is urine mixed with broken-down and disintegrated blood-cells; and instead of being regarded as a true hemorrhage that is to be checked by hæmostatics, it should be regarded as an effort of nature to throw off from the system a foreign substance that is hurtful to it.

The general principles of treatment are to avoid all remedies that tend by their action to depress the already prostrated vital functions; to restrain vomiting, and assuage thirst by the free use of ice; and to guard against another chill by the generous use of quinine. In addition to this, the most successfully treated cases were those in which lime-juice and iron were freely used; the lime-juice because its beneficial effects are so well known in scurvy, a kindred disease, and iron as furnishing a ready means of converting white into red corpuscles, and thereby restoring the loss occasioned by the death of so large a portion of the blood.

It is the experience of all the physicians with whom I have conversed, who have had opportunities of judging, that calomel in this disease is not borne at all, many attributing their unvarying want of success, among their first patients, to its use. As to the advisability of giving morphine, or any of the preparations of opium, until the severity of the disease shall have passed, I am not prepared to speak. Dr. T. Jones, who has seen more of this disease than any of us, and whose success in its treatment is very gratifying, is an advocate for its use; but I am inclined to think that I have seen mischief follow its administration, in checking the feeble eliminative process going on at the time.

I should perhaps mention, before closing, as another peculiarity of this disease, that its ravages have been almost exclusively confined to what is regarded as the healthiest portion of the country—the pine woods.

Albany, Georgia, May 14th, 1872.

